# Depression is associated with efavirenz-containing treatments in newly antiretroviral therapy initiated HIV patients in Ecuador

**DOI:** 10.1186/s12981-020-00303-1

**Published:** 2020-07-29

**Authors:** Alejandro Checa, Alberto Castillo, Malena Camacho, William Tapia, Isabel Hernandez, Enrique Teran

**Affiliations:** 1 Unidad de Atención Integral VIH/SIDA, Hospital Eugenio Espejo, Ministerio de Salud Publica del Ecuador, Quito, Ecuador; 2 Colegio de Ciencias de la Salud, Universidad San Francisco de Quito, Diego de Robles s/n y Pampite, Quito, Ecuador; 3 Facultad de Enfermeria, Pontificia Universidad Católica del Ecuador, Quito, Ecuador

**Keywords:** Efavirenz, Depression, Newly diagnose, HIV, Hamilton Rating Scale for Depression

## Abstract

**Background:**

It is well known that people living with HIV (PLWH) is in higher risk for the development of depression and it has also been suggested that the use of efavirenz into the antiretroviral regimens increases even that risk.

**Objective:**

To evaluate the effect of efavirenz-containing antiretroviral regimens on the development of depression in newly ART initiated HIV patients in Ecuador.

**Methods:**

In a prospective cohort study from June 2016 to May 2017, all newly HIV diagnosed patients at the HIV/AIDS Unit of the Hospital Eugenio Espejo in Quito, Ecuador were evaluated using the Hamilton Rating Scale for Depression followed by a second assessment 8–12 weeks after antiretroviral therapy containing efavirenz was initiated.

**Results:**

A total of 79 patients, mainly males younger than 35 years were studied. Majority of them were on TDF/FTC/EFV. Initial score in Hamilton Rating Scale revealed that less than 30% had no depression symptoms while almost 40% had mild depression. However, in the second assessment, 22.6% of the subjects had a score in the Hamilton Rating Scale compatible with severe or very severe depression (RR 1.58, 95% CI 1.09 to 2.28; p = 0.05).

**Conclusion:**

In our cohort study, depression was much higher in patients on Efavirenz-containing treatments. Therefore, assessment for depression must be essential as part of follow-up in these patients.

## Background

In Ecuador, according to the Ministry of Public Health, in 2018 there were 43,887 Persons Living With Human immunodeficiency virus (PLWH), the majority of those were men (70.6%) and between 20 and 44 years old (72.5%). Within recent years, the Ecuadorian government has increased funding to provide nationwide treatment to human immunodeficiency virus (HIV) positive individuals towards reaching the goals of 90% of HIV diagnosed individuals receiving treatment and 90% of individuals having viral suppression, but unfortunately only 57% of them are receiving combination antiretroviral therapy (cART) and only 51% have achieved viral suppression [1, 2].

International HIV treatment guidelines recommend first-line use of two nucleoside reverse transcriptase inhibitors (NRTIs) with an non-NRTI (NNRTI), a boosted protease inhibitor (PI) or an integrase inhibitor in order to achieve sufficient HIV RNA suppression [3]. Efavirenz (EFV), a benzoxamine, has long been considered the NNRTI par excellence, forming part of therapeutic combinations together with lamivudine/zidovudine, abacavir/lamivudine, tenofovir/emtricitabine [4]. However, since its approval, psychiatrics’ side effects has consistently been reported in 25–40% of patients on efavirenz [5] making these the drug’s most frequent side effect and is the main reason for discontinuing/switching therapy [6, 7].

Worldwide, mood and anxiety disorders, especially depression, are common among PLWH [8]. Currently, 39% of HIV patients are reported to suffer from depression, and patients treated with EFV experiencing psychiatric symptoms such as mania, depression, suicidal thoughts, psychosis, and hallucinations ranging from 61 to 90% [9]. Moreover, in a recent study in Ecuador [10], depression among PLWH (11.9%) was significantly higher than in the general population (4.6%).

Thus, the aim of this study was to evaluate the effect of efavirenz containing antiretroviral regimens on the development of depression in newly ART initiated HIV patients in Ecuador.

## Subjects and methods

This was a cohort prospective study conducted between June 2016 and May 2017 at the HIV Unit in the Hospital Eugenio Espejo in Quito, Ecuador. This hospital is one of the biggest in Ecuador and is responsible to provide attention for at least 10% of PLWH nationwide. Study conduction was authorized by the hospital review board and informed consent was obtained from each subject.

All new patients attending to the HIV clinic in the above mentioned period with a diagnose of HIV in the last 6 months confirmed by a positive blood analysis performed at the national reference laboratory of the Ministry of Health were invited to participate in this study. Then, after signed the consent form, each was evaluated using the Hamilton Rating Scale for Depression [11], which briefly used five categories: no depression symptoms (< 7 points), and then mild (8–13 points), moderate (14–18 points), severe (19–22 points) and very severe (> 23 points) depression. The Spanish version of the Hamilton Rating Scale has been previously validated [12].

The assessment with the Hamilton Rating Scale was performed before patients initiated the combined ART containing efavirenz and then between 8 and 12 weeks after the use of the combined ART including efavirenz. Subjects with previous history of depression, psychiatric disease or not able to receive efavirenz were excluded.

Data captured was entered into an electronic database and descriptive plus inferential statistics (Fisher exact test and relative risk with 95% confidence interval, using approximation of Katz) were performed in GraphPad InStat version 3.00. In all cases a p < 0.05 was considered as significant.

## Results

A total of 79 subject were recruited, mainly males (86.1%) and with an average age of 28.0 ± 8.3 years old (Table 1). Initial treatment was TDF/FTC/EFV (91.1%), ABC/3TC/EFV (7.6%), or AZT/3TC/EFV (1.3%). Of those 79 patients, eight (10.1%) did not completed the second assessment and therefore were excluded from further analysis. Mean follow up of these subjects was 12 weeks (range 2 to 62 weeks), in the initial Hamilton Rating Scale assessment, before treatment, only 29.6% (n = 21) patients had no depression. From those already positive for depression, the vast majority (40.8%, n = 29) had mild depression, followed by a 19.7% (n = 14) with moderate depression. There was only one subject with very severe depression and six patients (8.5%) with severe depression.

**Table 1:** Characteristics of the HIV positive subjects included in the study

	Males	Females
Age	28.3 ± 8.8	30.2 ± 6.0
< 20	8	0
21–25	21	2
26–30	16	4
31–35	5	2
> 35	11	2
Occupation		
Student	12	2
Housekeeping	1	5
Unemploy	14	1
Professional (lawyer, engineer, other)	6	1
Hairdresser	8	1
Handworker	4	0
Artist	2	0

**Fig. 1:**
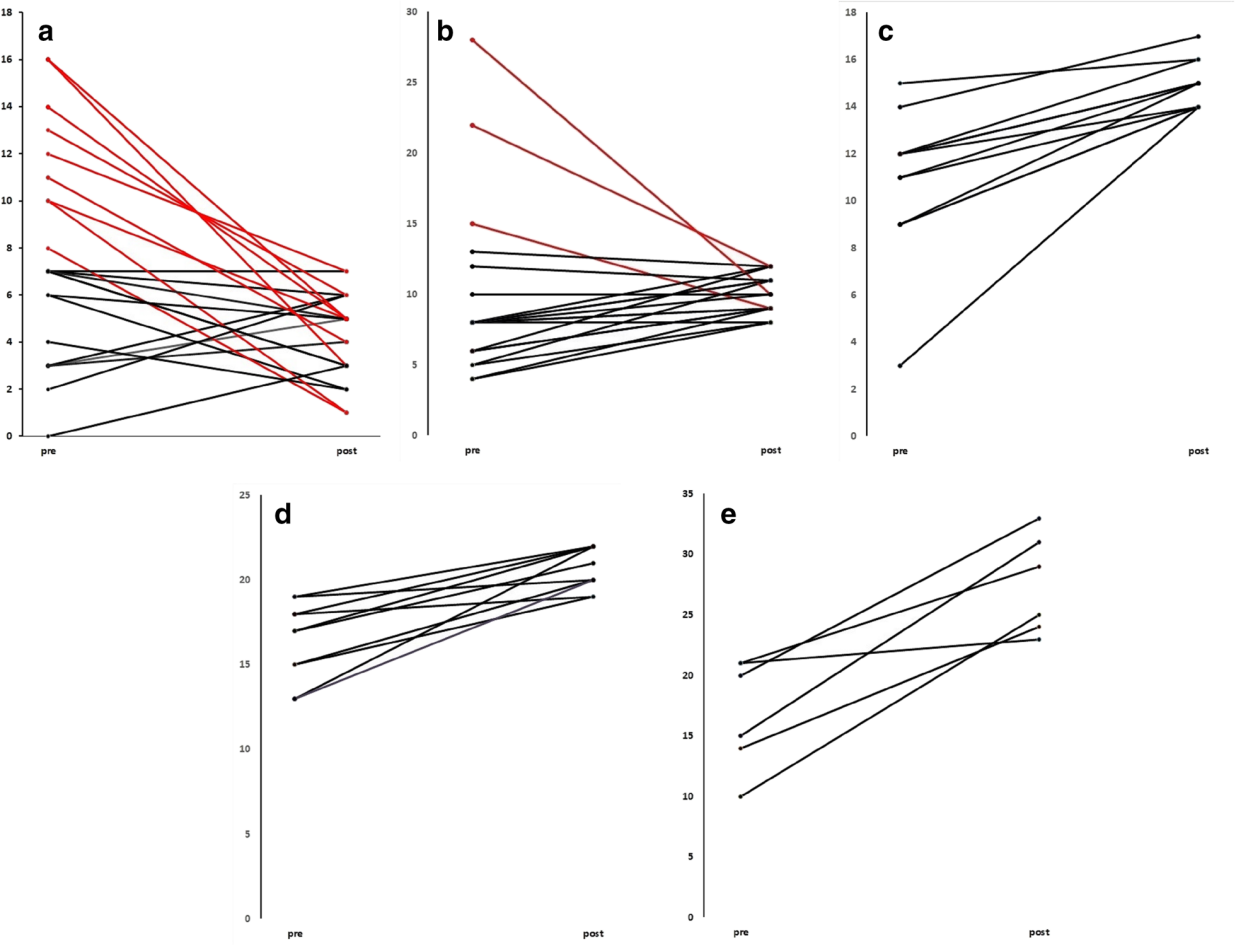
Before and after plots of the patients with depression assessed by the Hamilton Rating Scale. **a** No depression, **b** mild, **c** moderate, **d** severe and **e** very severe depression

In the second assessment, a median of 69 days (IC 95% 58 to 80 days) after treatment with combined ART including efavirenz was initiated, 22 (31%) subjects had a score negative for depression in the Hamilton Rating Scale. However, only 13 of them (59.1%) were initially without depression. Nine patients improve their score in the Hamilton Rating Scale, from mild (n = 6) or moderate depression (n = 3, Fig. 1a).

Mild depression was found in a slightly lower number of patients (n = 21; 29.6%) in comparison to the initial assessment (p = 0.4). However, of those less than half (n = 11) were previously in the same category according to the Hamilton Rating Scale. From the remaining ten subjects, three improved their initial condition, i.e. one had initially very severe depression and the other two had severe depression, while the in the other seven patients their score in the Hamilton Rating Scale increase, suggesting their condition worsened (RR 0.72, 95% CI 0.48 to 1.08; Fig. 1b).

Regarding subjects with moderate depression in the second Hamilton Rating Scale assessment, it was present in twelve patients (16.9%), but only two of them were previously in the same category. All other subjects (n = 10) with moderate depression previously had mild depression (RR 0.90, 95% CI 0.56 to 1.44; Fig. 1c). Cases of severe depression by the Hamilton Rating Scale in the second assessment increased to ten patients (14.1%), six of them had moderate depression and two mild depression previously (RR 1.16, 95% CI 0.75 to 1.78; Fig. 1d).

Finally, cases of very severe depression detected by Hamilton Rating Scale in the second assessment showed a significant increase (p = 0.02) with 6 cases (8.5%), three were previously in the severe group, two from the moderate group and one from mild depression (RR 2.14, 95% CI 1.72 to 2.65; Fig. 1e).

**Fig. 2:**
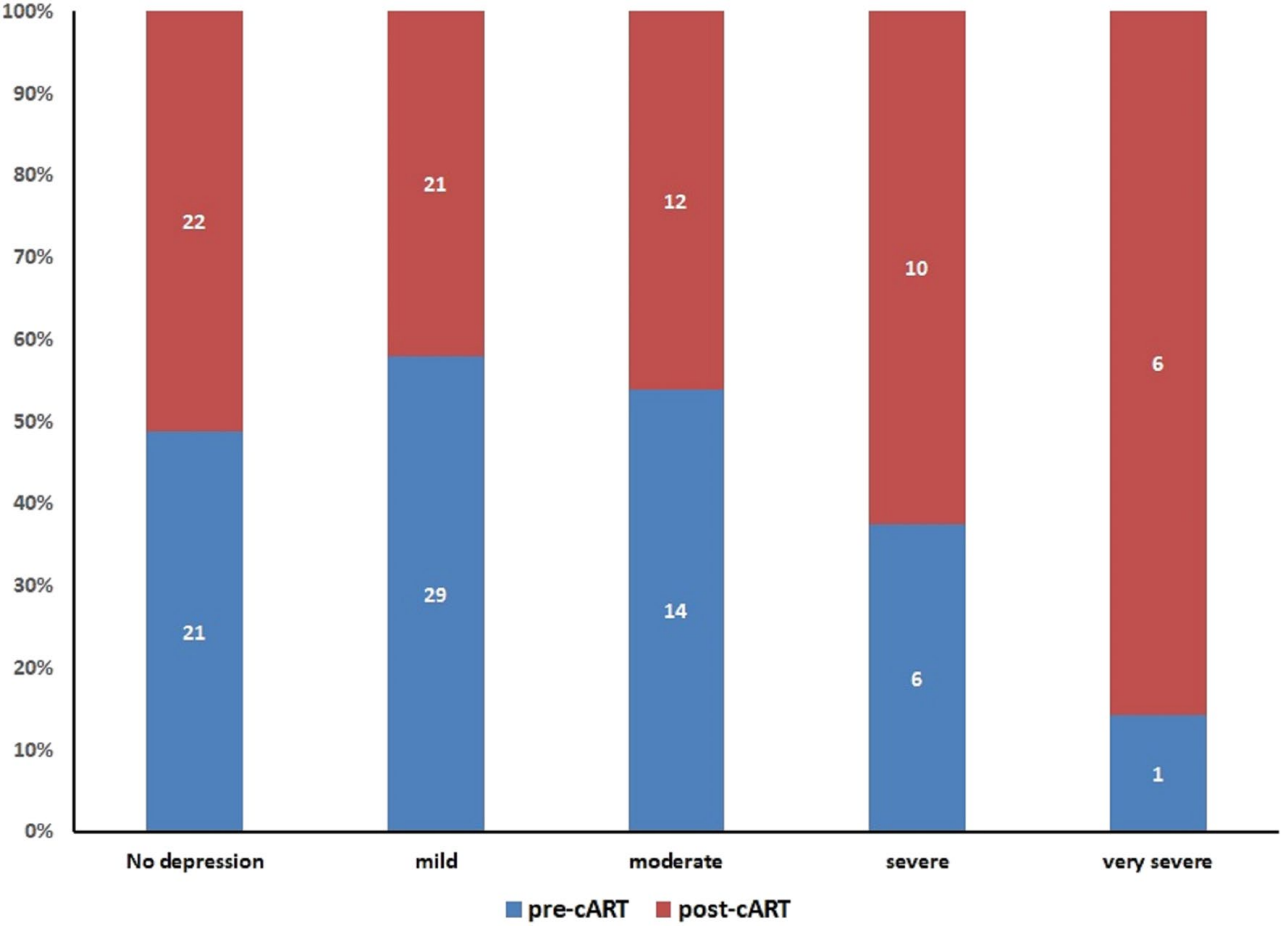
Distribution of the HIV positive subjects to the different categories based on the Hamilton Rating Scale before and after initiating combined antiretroviral therapy containing efavirenz

Finally, in patients positive for HIV the use of combined ART including efavirenz was associated to a significative increase in the number of cases with severe or very severe depression; (RR 1.58, 95% CI 1.09 to 2.28; p = 0.05; Fig. 2.

## Discussion

In the present study, it was found that vast majority of PLWH attended to the HIV Clinic at Hospital Eugenio Espejo in Quito, Ecuador had any degree of depression, much more than previously reported [10]. In this sense, it is important to mention that mental health is a usually neglected area among health care services in Ecuador which leads to failure to diagnose physiological and psychiatric problems among the general population not to mention among PLWH.

Most of the subjects with depression in this study worsen, consistently with previous evidence that EFV increases the activity of the enzyme tryptophan-2-3-dioxygenase (TDO) that has an inverse relationship with the levels of serotonin (5-HT) in the brain [13]. It is consistent with other studies showing the number of CNS effects attributed to EFV were almost double that of the various antiretroviral with which it has been compared [6, 7], and also with reports that CNS symptoms tends to improve or even to disappear after EFV discontinuation [14].

There were also few cases were depression improved after a regimen containing EFV, something that also has been reported eventually [5], but not fully explained, other studies did show a significant reduction in CNS adverse events after switching from efavirenz to another antiretroviral agent [15–17].

While we recognize that screening tools used for depression could have increased the number of patients diagnosed, we feel that the criteria used allowed us to select for the most clinically significant cases. Also, another limitation was that subjects were recruited only in one center, although one of the biggest, and there was no control group due to national guidelines. Anyway, our results support that EFV-containing regimens increase both, the frequency and severity, of depression cases among PLWH in Ecuador.

## Conclusion

In our cohort study, in PLWH in Ecuador, the use of combined ART containing efavirenz is associated with depression. Therefore, assessment and monitoring for depression must be essential in the follow-up of these patients.

## Data Availability

The datasets used and/or analyzed during the current study are available from the corresponding author on reasonable request.
